# Supplementary material for: Can MDCT Enhancement Patterns Be Helpful in Differentiating Secretory from Non-Functional Adrenal Adenoma?
Source: Medicina (Kaunas). 2023 Dec 29;60(1):72. doi: 10.3390/medicina60010072 (PMC10819253; doi:10.3390/medicina60010072)
Supplement: Supplementary file 1 [file medicina-60-00072-s001.zip › supplementary data.pdf]

Morphological characteristics of adrenal masses were evaluated in the native MDCT phase with no statistically significant differences between MDCT morphological parameters in relation to secretory activity (Table S1).

Table S1. MDCT morphological characteristics of adrenal masses obtained in the unenhanced phase in relation to secretory activity

		Secretory activity		
MDCT morphological features Native phase		Nonfunctional masses	Secretory masses	Total
Shape	Regular	15	10	25
	Lobulated	13	11	24
	Irregular	1	0	1
	Total	29	21	50
Margins	Smooth	12	7	19
	Lobulated	15	12	27
	Irregular	2	2	4
	Total	29	21	50
Calcification	Absent	20	19	39
	Present	9	2	11
	Total	29	21	50
Septa	Absent	27	21	48
	Present	2	0	2
	Total	29	21	50
Necrosis	Present	9	5	14
	Absent	20	16	36
	Total	29	21	50
Lipid Component	Lipid-poor	9	13	19
	Lipid-rich	20	8	31
	Total	29	21	50
Cystic degeneration	Absent	26	18	44
	Present	3	3	6
	Total	29	21	50

Morphological characteristics of adrenal adenoma were evaluated in the native MDCT phase with no statistically significant differences between MDCT morphological parameters in relation to secretory activity (Table S2).

Table S2. MDCT morphological characteristics between secretory and nonfunctional adenoma obtained in the unenhanced phase in relation to secretory activity

		Secretory activity		
MDCT morphological features Native phase		Nonfunctional adenoma	Secretory adenoma	Total
Shape	Regular	15	6	21
	Lobulated	15	9	22
	Irregular	1	0	1
	Total	29	15	44
Margins	Smooth	12	3	15
	Lobulated	15	10	25
	Irregular	2	2	4
	Total	29	15	44
Calcification	Absent	20	13	33
	Present	9	2	11
	Total	29	15	44
Septa	Absent	27	15	42
	Present	2	0	2
	Total	29	15	44
Necrosis	Present	9	4	13
	Absent	20	11	31
	Total	29	15	44
Lipid Component	Lipid-poor	11	8	18
	Lipid-rich	20	7	27
	Total	29	15	44
Cystic degeneration	Absent	26	13	39
	Present	3	2	5
	Total	29	15	44
